# Upadacitinib for refractory Behçet’s disease with myelodysplastic syndrome and trisomy 8/9: a case report and mechanistic insights

**DOI:** 10.3389/fimmu.2025.1609884

**Published:** 2025-05-27

**Authors:** Yukai Wang, Feng Tian, Hui Li

**Affiliations:** Department of Gastroenterology, Shengjing Hospital of China Medical University, Shenyang, Liaoning, China

**Keywords:** Behçet’s disease, myelodysplastic syndrome, trisomy 8, upadacitinib, JAK-STAT pathway, refractory disease

## Abstract

**Background:**

Behçet’s disease (BD), a multisystemic inflammatory disorder with genetic predisposition, is frequently complicated by myelodysplastic syndrome (MDS), particularly in cases harboring trisomy 8. Patients with refractory BD-MDS exhibit poor responses to conventional therapies, including glucocorticoids and TNF-α inhibitors, underscoring the need for novel therapeutic strategies. Janus kinase (JAK) inhibitors, which target cytokine-driven inflammation, represent a promising approach; however, clinical evidence in genetically complex BD-MDS cases remains limited.

**Case presentation:**

We report a 29-year-old female with refractory intestinal BD, MDS, and dual trisomy 8/9, who presented with recurrent ulcers, thrombocytopenia, and ileocolonic resection due to perforation. Despite sequential therapies (thalidomide, prednisolone, and infliximab), disease progression persisted. Initiation of upadacitinib (45 mg/day), a selective JAK1 inhibitor, resulted in symptom resolution within one week and complete mucosal healing confirmed by colonoscopy at three months. Dose reduction to 15 mg/day led to disease relapse, while maintenance at 30 mg/day sustained remission over 12 months.

**Methods:**

Immunohistochemical (IHC) analysis of intestinal specimens from the patient and three additional BD cases revealed robust phosphorylation of JAK1 and STAT3 in mucosal epithelium, stroma, and inflammatory infiltrates, particularly within occluded submucosal vessels. These findings mechanistically implicate JAK-STAT hyperactivation in BD-associated vascular pathology.

**Conclusion:**

This study highlights the efficacy of upadacitinib in managing refractory BD with MDS and dual trisomy 8/9, a genetically complex phenotype. The dose-dependent response underscores the importance of tailored dosing strategies. Our mechanistic data further support JAK inhibition as a viable therapeutic alternative for TNF-α inhibitor-resistant BD. These results warrant validation through randomized controlled trials to optimize therapeutic protocols for similar high-risk populations.

## 1 Introduction

Behçet’s disease (BD), a chronic multisystemic inflammatory disorder characterized by recurrent mucocutaneous ulcers, ocular inflammation, and vascular involvement, exhibits a strong genetic predisposition, particularly among populations along the historic Silk Road (1). While its pathogenesis remains incompletely elucidated, dysregulated immune responses involving Th1/Th17 polarization and cytokine-driven inflammation are central to disease progression (1). Emerging evidence highlights a distinct clinical subset of BD complicated by myelodysplastic syndrome (MDS), a hematologic malignancy marked by cytopenias and clonal chromosomal abnormalities (2). Notably, trisomy 8—a karyotypic aberration observed in 7–9% of primary MDS cases—is present in over 80% of BD-MDS patients, suggesting a unique pathophysiologic interplay (3–5). These patients often present with severe gastrointestinal (GI) involvement, including deep ileocecal ulcers requiring surgical intervention, alongside hematologic abnormalities such as macrocytic anemia and thrombocytopenia (6–8). Despite aggressive management, BD-MDS patients exhibit poor responses to conventional therapies, including corticosteroids and tumor necrosis factor-alpha (TNF-α) inhibitors, with mortality rates exceeding 25% (6, 9). This underscores the urgent need for novel therapeutic strategies targeting the underlying molecular drivers of refractory disease.

The JAK-STAT signaling pathway, a critical mediator of cytokine signaling, has emerged as a key contributor to BD pathogenesis. Upregulated JAK1 and STAT3 activation in monocytes and T cells drives the production of pro-inflammatory cytokines (e.g., IL-6, IL-17, IFN-γ), perpetuating systemic inflammation and mucosal damage (10). Preclinical and clinical studies suggest that selective JAK inhibitors, such as upadacitinib, may attenuate Th1/Th17 responses and induce remission in refractory BD (10). However, evidence supporting their efficacy in genetically complex BD-MDS cases, particularly those with dual trisomy 8/9, remains scarce.

Here, we present a case of refractory intestinal BD complicated by MDS and dual trisomy 8/9, a rare karyotypic profile associated with heightened treatment resistance. Through immunohistochemical analysis of intestinal specimens, we further elucidate the role of JAK-STAT hyperactivation in BD-associated vascular pathology. Our findings highlight the therapeutic potential of upadacitinib in this challenging patient population and provide mechanistic rationale for JAK inhibition as a targeted strategy in TNF-α inhibitor-resistant BD.

## 2 Case presentation

**Figure 1:**
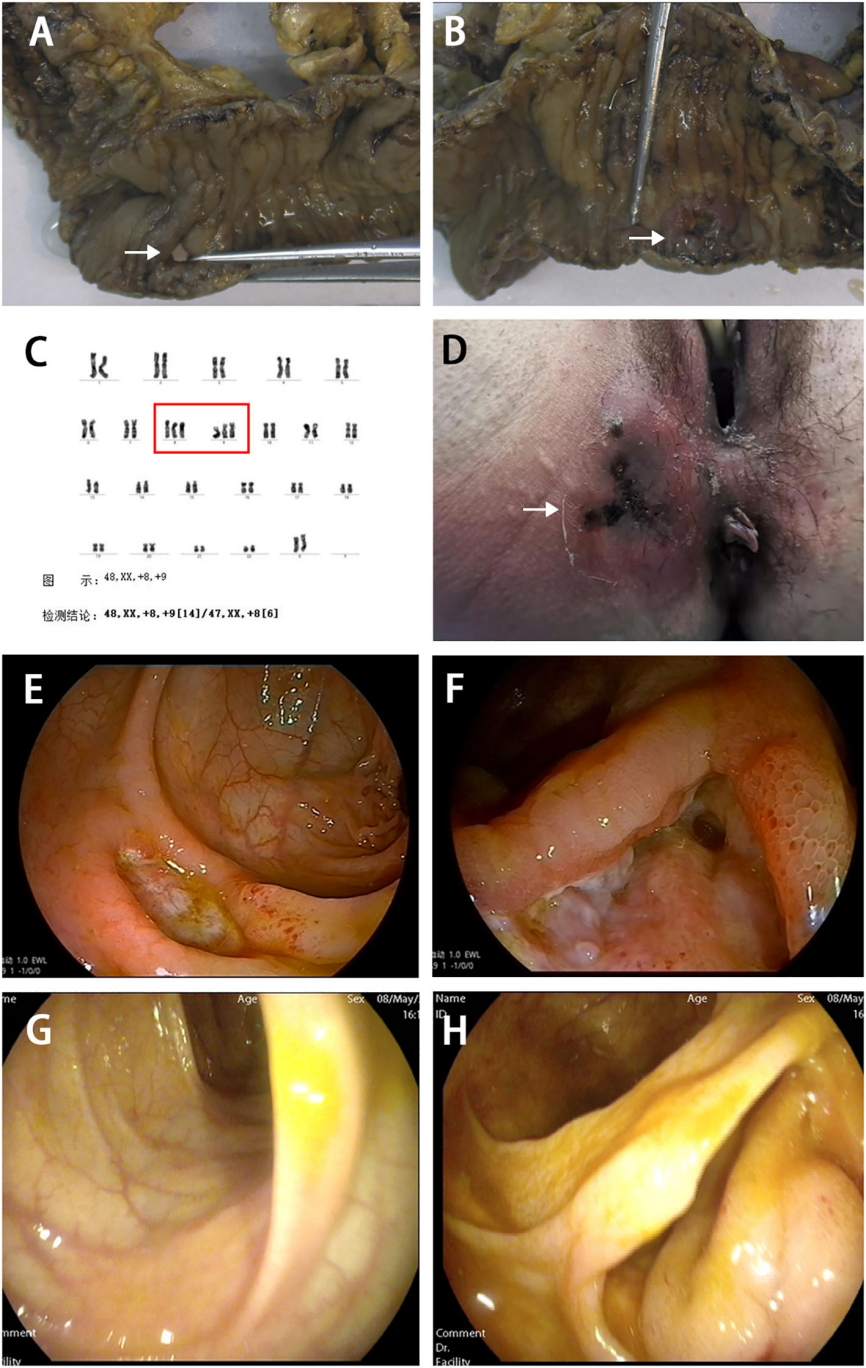
Surgical resection of intestinal specimens and clinical findings in this patient. **(A)** Resected intestinal specimen showing ileal perforation (indicated by white arrow). **(B)** Deep ulcer observed in the terminal ileum (indicated by white arrow). **(C)** Bone marrow chromosome examination showing trisomy 8 and 9 (indicated by red box). **(D)** In August 2021, the patient developed a perianal abscess with necrotizing fasciitis (indicated by white arrow). Colonoscopy showed ulcer at the descending colon **(E)** and the anastomotic site **(F)**. After three months of treatment with upadacitinib, mucosal healing was observed in the descending colon **(G)** and the anastomotic site **(H)** after three months of treatment with upadacitinib.

A 29-year-old female patient presented with a two-year history of recurrent oral ulcers and a one-year history of genital ulcers and thrombocytopenia. In August 2019, she was admitted due to acute small bowel perforation. Emergency laparotomy revealed a perforation 30 cm proximal to the ileocecal valve, accompanied by a deep ulcer in the terminal ileum (**Figures 1A, B**). Segmental resection of the ileum, ileocecal region, and ascending colon was performed, followed by end-to-end anastomosis. Histopathological examination of the resected specimen revealed submucosal vascular dilation, intimal hyperplasia of small arteries, and thrombi within the arterial and venous lumina. Postoperative diagnosis confirmed BD. A bone marrow biopsy performed due to persistent thrombocytopenia. Bone marrow biopsy was performed due to persistent thrombocytopenia. The biopsy revealed increased myeloblasts (1.6%), active erythroid hyperplasia (mainly intermediate/late-stage erythroblasts), and significant nuclear/cytoplasmic abnormalities in erythrocytes, with pathological cells comprising 38% of the erythroid lineage. Flow cytometry showed 1.1% CD34-positive immature myeloid cells. Karyotype analysis indicated trisomy 8 and trisomy 9 (**Figure 1C**). Although next-generation sequencing was recommended, the patient declined due to cost. The diagnosis of MDS was confirmed based on these findings. The patient was classified as International Prognostic Scoring System (IPSS) intermediate-2 (11) and revised IPSS (IPSS-R) high-risk (12). However, the patient refused allogeneic hematopoietic stem cell transplantation due to associated risks and costs. Initial treatment with thalidomide (50 mg/day) was discontinued after six months due to peripheral neuropathy. In July 2020, a recurrent oral ulcer prompted the initiation of prednisolone (40 mg/day), which was gradually tapered. However, disease relapse occurred in March 2021 (prednisone 10 mg/day), characterized by new oral ulcers and right mid-abdominal pain. Colonoscopy revealed an anastomotic ulcer, prompting infliximab induction (5 mg/kg) at weeks 0, 2, 6, and 8, followed by maintenance therapy every 8 weeks. A follow-up colonoscopy at three months confirmed ulcer healing, and maintenance infliximab therapy (every 8 weeks) was initiated.

In August 2021, the patient developed a perianal abscess with necrotizing fasciitis (**Figure 1D**). She recovered well after surgical drainage and continued regular infliximab therapy. Despite regular infliximab, disease activity recurred in December 2022, marked by oral ulcers, abdominal pain, and elevated CRP. Infliximab dosing was escalated to every 4 weeks, yet symptoms progressed. By February 2024, colonoscopy revealed recurrent anastomotic and descending colon ulcers (**Figures 1E, F**). Infliximab was discontinued, and upadacitinib (45 mg/day) was initiated.

**Figure 2:**
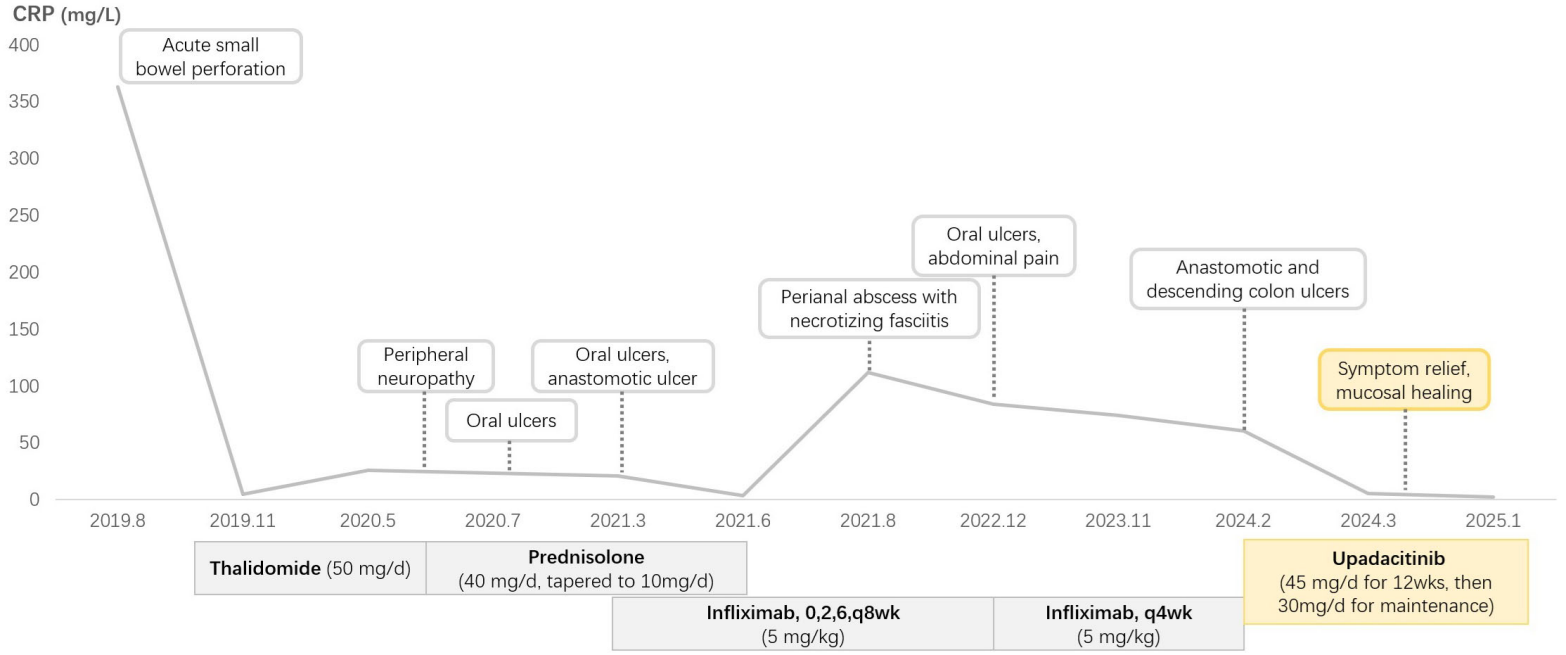
Clinical course of this patient.

Clinical improvement was noted within one week, with complete resolution of abdominal pain. A repeat colonoscopy performed three months after the initial procedure demonstrated mucosal healing (**Figures 1G, H**). The platelet count remained subnormal (95×10^9/L; reference range 150–450×10^9/L), but the CRP level normalized, and no active ulcers were observed. Dose reduction to 15 mg/day resulted in recurrent oral ulcers, necessitating maintenance therapy with 30 mg/day. At the last follow-up (March 2025), the patient remained asymptomatic on upadacitinib 30 mg/day. The patient’s clinical course is shown in **Figure 2**.

**Figure 3:**
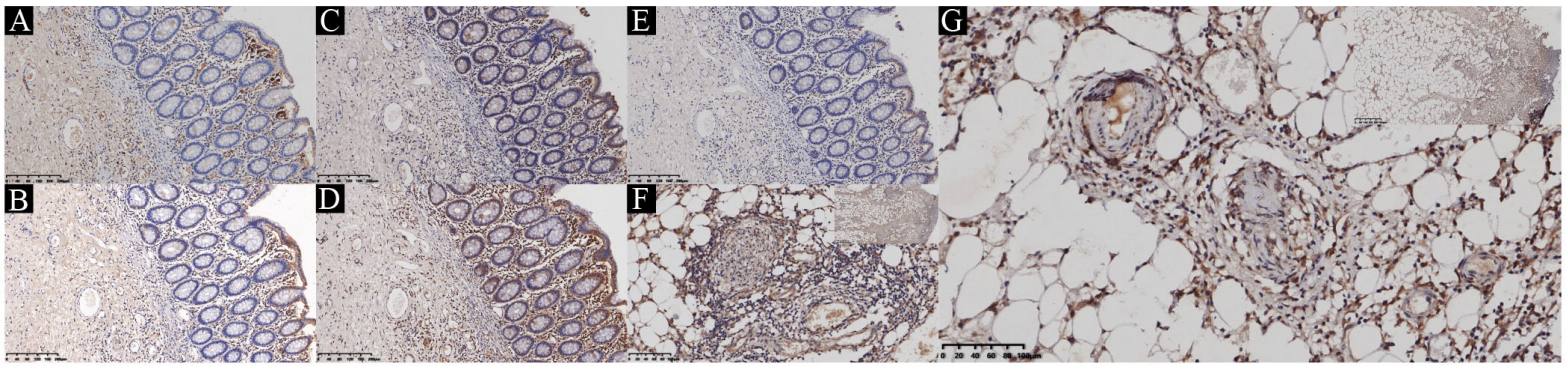
Immunohistochemical staining of JAK-STAT in the ileum. **(A)** Phosphorylated JAK-1. **(B)** Phosphorylated JAK-2. **(C)** Phosphorylated JAK-3. **(D)** Phosphorylated STAT1. **(E)** Phosphorylated STAT3. **(F)** Representative image of immunohistochemical staining of phosphorylated JAK-1 in typical vasculitic lesions. **(G)** Representative image of immunohistochemical staining of phosphorylated STAT3 in typical vasculitic lesions.

Based on these clinical observations, we performed IHC staining on surgically resected intestinal specimens from the patient. The study was approved by the Ethics Committee of the Shengjing Hospital of China Medical University (approval number: 2024PS1286K). The expression of phosphorylated JAK-1, JAK-2, JAK-3, STAT1, and STAT3 was detected in the mucosal epithelium, mucosal stroma, intrinsic glands, small blood vessels, and peripherally infiltrating inflammatory cells within the submucosal layer (**Figures 3A-E**). Notably, phosphorylated JAK-1 and STAT3 exhibited strong expression in the lumen of occluded small vessels in the submucosal layer, smooth muscle cells, and peripherally infiltrating inflammatory cells (**Figures 3F, G**). These findings suggest that upadacitinib may represent a promising therapeutic option for patients with Behçet’s disease (BD) who are refractory to TNF-α inhibitors.

## 3 Literature review and discussion

The present case illustrates the sustained efficacy of upadacitinib, a selective JAK1 inhibitor, in achieving remission for refractory intestinal BD complicated by MDS and dual trisomy 8/9—a rare and genetically complex phenotype. MDS is increasingly recognized for its association with autoimmune and autoinflammatory manifestations, which significantly influence clinical trajectories and outcomes. These manifestations, ranging from mucocutaneous involvement to systemic inflammatory syndromes, are mechanistically linked to somatic mutations (e.g., *RUNX1*, *TP53*, *TET2*, *ASXL1*) and epigenetic alterations that disrupt immune cell homeostasis and promote dysregulated cytokine signaling (13). Aberrant innate immune activation, mediated by NLRP3 inflammasome hyperactivity and oxidative stress, further amplifies inflammatory cascades (14, 15). Additionally, dysfunction within the bone marrow microenvironment, including stromal cell abnormalities, contributes to chronic inflammation and hematopoietic failure (16). Notably, somatic mutations such as *RUNX1* have been implicated in atypical inflammatory presentations preceding MDS progression, underscoring the interplay between clonal hematopoiesis and immune dysregulation (17). While genetic profiling was unavailable in our case due to patient refusal, the observed dual trisomy 8/9—a karyotype associated with aggressive disease phenotypes—likely exacerbates treatment resistance through undefined molecular mechanisms. This highlights the critical need for comprehensive genomic analyses in similar cases to elucidate genotype-phenotype correlations and identify actionable therapeutic targets.

Emerging evidence supports JAK inhibition as a promising strategy for cytokine-driven inflammatory disorders, particularly in cases refractory to conventional biologics. By reviewing the published literature associated with JAK inhibitors in treatment of BD, we identified 13 studies or case reports on BD treated with JAK inhibitors, including 3 pilot studies, 2 case series, and 8 case reports, totaling 69 cases. Among these, 32 patients received tofacitinib (18–23), 30 received baricitinib (24–26), and 7 received upadacitinib (27–32) (**Table 1**).

**Table 1:** Summary of JAK Inhibitor Therapies in Behçet's Disease: Clinical Features, Treatments, and Outcomes.

Jak inhibitor	Author(year)	Country/region	Article type	Age(y)/Sex	Clinical features and complications	Previous treatment	Dosage	Follow-up (months)	Outcome	Adverse events	Ref.
Tofacitinib	Liu et al. (2020)	China	Pilot study	42(30,48)/7M, 6F	Vascular/cardiac involvement (n=5), gastrointestinal inolvement (n=6), articular (n=2)	CS, AZA,CTX,LEF, COL, TCZ, THD, SASP, MMF, TAC, IFX, ADA, GOL	5 mg BID	8 (IQR 5.5–19)	Overall BDCAF score improved significantly. Patients with vascular/cardiac and articular involvement achieved remission. ESR and CRP levels decreased significantly.	Herpes zoster (n=2)	(11)
	Wang et al. (2022)	Taiwan	Case report	27/Female	Arthritis, orogenital ulcerations, papulopustular lesions, anterior uveitis	CS, COL, MTX, AZA	11 mg/day	>6	Disease remission, sparing the use of corticosteroids	No adverse events reported	(12)
	Zou et al. (2022)	China	Retrospective study	40(31,43)/3M, 10F	Intestinal involvement (ileocecal ulcers, small intestine ulcers, etc.)	CS, IFX, AZA, CSA, ETN, ADA, HCQ, CTX, MTX	5 mg BID (n=11), 5 mg/day (n=2)	10.1±1.9	Clinical remission in 8/13, mucosal healing in 6/11, significant reduction in DAIBD scores and CRP levels	No serious adverse events reported	(13)
	Zhao et al. (2022)	China	Case series	67/M, 20/F, 49/F, 31/F	Intestinal ulcers (ileocecal, small intestine, etc.), abdominal pain	CS, IFX, AZA, SASP, THD, CTX, MTX	5 mg BID or 5 mg/say	10-33	Disease remission, mucosal healing, reduction in inflammatory markers	No adverse events reported	(14)
	Lin et al. (2023)	China	Case report	34/Male	Oral ulcers, genital ulcers, skin leison, parastomal ulcers, articular involvement	CS, THD, IFX, CTX, LEF, MTX	5 mg BID	12	Parastomal ulcers healed, reduction in inflammatory markers	No adverse events reported	(15)
	Rao et al. (2024)	India	Retrospective study	11/NS	NS	NS	NS	NS	Eleven-patients received biologics(anti-TNF-α) and JAK inhibitors to treat severe organ involvement.	NS	(16)
Baricitinib	Liu et al. (2023)	China	Pilot study	34(29,36)/6M, 7F	Intestinal ulcers (ileum, ileocecal junction, colon), anastomotic ulcers, fistulas, colostomy, bowel obstruction	TOF, CTX, MMF, TAC, SASP, LEF, AZA, COL, THD, IFX, ADA, GOL, INH, RPT, CS, 5-ASA, MMF, LEF, CsA	2 mg/day (escalated to 4 mg/day in 4 cases)	11 (IQR 9–14)	Complete remission in 76.92% (10/13), mucosal healing in 66.7% (6/9), significant reduction in DAIBD scores and CRP levels	No thrombotic events or severe infections observed	(17)
	Wang et al. (2023)	China	Pilot study	34(28,46)/12M, 5F	Vascular involvement (venous lesions, arterial lesions, cardiac involvement)	CTX, CO, THD, LEF,MMF, MTX, AZA, CsA,TAC	2 mg/day	10.7±5.3	Complete remission in 88.2% (15/17), significant reduction in ESR, hsCRP, BDCAF scores	No serious adverse events observed	(18)
	Ito et al. (2024)	Japan	Case report	61/Female	Oral ulcers, genital/rectal ulcers, rheumatoid arthritis, peristomal ulceration	CS, ADA, COL, CsA, SASP	NS	36	RA improved with Baricitinib in 1 week; peristomal PG and genital/rectal ulcers did not relapse after upadacitinib for 3 years	No adverse events reported	(19)
Upadacitinib	Tao et al. (2024)	China	Case series	Adolescent girl, man in his thirties	Oral and orogenital ulcers, macular edema, panuveitis, skin erythema	CS, MTX, ADA, CsA, MMF	15 mg/day	5-9	Improved visual acuity, controlled intraocular inflammation, disappearance of macular edema	Mild leukopenia and transaminitis in the female patient	(20)
	Kraev et al. (2024)	Bulgaria	Case report	42/Female	Ankylosing spondylitis, sacroiliitis, panuveitis, oral and genital ulcers, ocular leison, hearing loss	CS ect.	15 mg/day	3	Significant alleviation of joint discomfort and mucosal ulcerations	No adverse events reported	(21)
	Vitale et al.(2024)	Egypt	Prospective study	57/Male	NS	NS	15 mg/day	NS	Complete ocular disease control was achieved at the last assessment	No adverse events reported	(22)
	Sha et al. (2024)	China	Case report	24/Male	Oral ulcers, intestinal ulcer(a single, well-defined large ulcer in the ileocecal region), anemia	CS, IFX, SASP, ADA	45 mg/day for 12 weeks, then 30 mg/day	12	Alleviation of abdominal pain, reduction in inflammatory markers, anemia corrected, ulcers healed at the 12-week follow-up	No severe adverse events observed	(23)
	Patel et al. (2025)	America	Case report	38/Male	Oral and perianal ulcers, articular involvement, acneiform pustules, tender nodules on the back, arms, legs, and genitals	CS, ADA, CS, RIT, MMF, SIR	15 mg/day	6	Alleviation of abdominal pain within 48 hours, resolved ulcers and joint pain by the tenth week	Bilateral pneumonia	(24)
	Ammoscato et al. (2025)	Italy	Case report	37/Female	Arthralgia/arthritis, anterior uveitis, de novo genital ulcers, skin lesions (including lesions on the hands, chest, knees, feet, and legs), neutrophilic dermatosis, inflammatory arthralgia, knee arthritis	CS, ETOR, ADA, GOL, IFX,CS, MTX	15 mg/day	7	Resolution of arthralgia/arthritis, remission of mucocutaneous lesions, sustained control of ocular and gastrointestinal symptoms within two weeks	No adverse events reported	(25)

ADA, Adalimumab; AZA, Azathioprine; BDCAF, Behçet's Disease Current Activity Form; COL, Colchicine; CTX, Cyclophosphamide; CS, Corticosteroids; CsA, Cyclosporine A; DAIBD, disease activity index of intestinal Behçet's disease; ETN, Etanercept; ETOR, Etoricoxib; GOL, Golimumab; HCQ, Hydroxychloroquine; IFX, Infliximab; INH, Isoniazid; LEF, Leflunomide; MTX, Methotrexate; MMF, Mycophenolate mofetil; NS, Not specified; RPT, Rifapentine; RIT, Rituximab; SIR, Sirolimus; SASP, Sulfasalazine; TAC, Tacrolimus; TCZ, Tocilizumab; THD, Thalidomide; TOF, Tofacitinib.

### 3.1 Role of the JAK-STAT pathway in Behçet’s disease

The JAK-STAT pathway is a critical signaling mechanism that mediates the effects of various cytokines and growth factors involved in immune responses, hematopoiesis, and cellular proliferation (33). This pathway comprises four members: JAK1, JAK2, JAK3, and TYK2, which are non-receptor tyrosine kinases that transmit signals from a wide range of cytokine receptors (34–36). The JAK-STAT pathway plays a significant role in the pathogenesis of BD. Studies have demonstrated upregulated JAK1 expression in CD14+ monocytes and CD4+ T cells of BD patients, with activation of the JAK/STAT signaling pathway in these cells (37). Moreover, the expansion of Th1 and Th17 cell subsets in BD patients is closely associated with the activation of the JAK/STAT pathway (38, 39). In BD, the JAK1/STAT3 signaling pathway is likely mediated by Th1/Th17-type cytokines, such as IL-2, IFN-γ, IL-6, IL-17, and IL-23, which are central to the inflammatory response and disease activity (40–43). Additionally, BD patients exhibit significantly higher STAT3 expression compared to healthy controls, both under unstimulated and stimulated conditions (37). A study in Han Chinese BD patients identified three single nucleotide polymorphisms (SNPs) in the JAK1 gene—rs2780815, rs310241, and rs3790532—that are significantly associated with BD susceptibility (44). These SNPs may increase the risk of BD by altering the expression or function of the JAK1 gene. Furthermore, no gene-gene interaction was found between the JAK1 gene and HLA-B51, indicating that JAK1 is an independent risk factor for BD (44). Our IHC findings revealed robust expression of phosphorylated JAK-1 and STAT3 in occluded submucosal vessels and inflammatory infiltrates, further supporting the therapeutic rationale for JAK inhibitors in BD.

### 3.2 JAK inhibitors in refractory BD

JAK inhibitors, including tofacitinib, baricitinib, and upadacitinib, have emerged as promising therapeutic options for BD (18, 30). Clinical studies and case reports consistently demonstrate their ability to alleviate symptoms, improve laboratory markers, and achieve endoscopic remission in cases that are refractory to other treatments.

Tofacitinib, a pan-JAK inhibitor primarily targeting JAK1 and JAK3, has yielded mixed results in gastrointestinal Behçet’s disease (BD). A pilot study of tofacitinib (5 mg twice daily) in 13 patients with refractory BD reported mucosal healing in only one of six patients with gastrointestinal involvement. At the same time, vascular and articular manifestations responded more favorably (18). Conversely, a retrospective study of 13 patients with gastrointestinal BD found clinical remission in nine patients and mucosal healing in six of 11 patients after a mean treatment duration of 10 months (20). A case series reported successful outcomes in four patients with severe refractory intestinal BD treated with tofacitinib, highlighting its ability to induce remission in cases where other therapies had failed (21). Tofacitinib also demonstrated efficacy in refractory parastomal ulcers, a condition resistant to conventional therapies (22).

Baricitinib, a selective JAK1/JAK2 inhibitor, has demonstrated promising efficacy in both intestinal and vascular Behçet’s disease (BD). In a pilot study of 13 patients with intestinal BD, baricitinib (2 mg daily, escalated to 4 mg in non-responders) achieved complete remission in 76.9% of patients and mucosal healing in 66.7%, with significant reductions in disease activity indices and CRP levels (24). Similarly, in vascular BD, 76.5% of patients achieved complete clinical and radiologic remission within three months of baricitinib therapy (25). Additionally, baricitinib has demonstrated efficacy in treating peristomal pyoderma gangrenosum associated with rheumatoid arthritis and Behçet’s disease (26).

Upadacitinib, a selective JAK1 inhibitor, has demonstrated rapid and sustained efficacy in patients with refractory BD, particularly in those resistant to TNF-α inhibitors. Six case reports (seven cases) highlight its ability to induce remission in patients with complex BD, including those with concurrent ankylosing spondylitis and macular edema (27, 28, 30–32). A recent prospective cohort study involving 12 patients with non-infectious inflammatory ocular diseases included one case of Behçet’s uveitis. This patient was treated with a combination of upadacitinib 15 mg/day and azathioprine 200 mg/day, achieving complete ocular disease control (29). In our case, considering the patient’s severe condition, including intestinal perforation, perianal abscesses, and refractory to TNF inhibitors, we chose an initial dose of 45 mg/day. Within three months on upadacitinib, the patient achieved rapid symptom resolution and mucosal healing. Unlike most reported cases where 15 mg/day sufficed, our patient required dose escalation to 30 mg/day for sustained control. This dosing strategy is similar to that reported by Sha et al., where upadacitinib was initiated at 45 mg/day for refractory intestinal BD in a patient who had previously failed corticosteroids and infliximab. The patient experienced rapid symptom improvement and mucosal healing within 12 weeks, after which the dose was tapered to 30 mg/day for maintenance therapy (30). The requirement for a higher initial dose in our patient may be attributed to the complexity of her disease profile, which included myelodysplastic syndrome with the extremely rare trisomy of chromosomes 8 and 9. This genetic abnormality likely contributed to the increased therapeutic challenge. Despite this, upadacitinib proved effective in managing her condition, highlighting its potential as a therapeutic option for complex BD cases.

### 3.3 Safety considerations

The safety data for JAK inhibitors primarily derive from patients with immune-mediated inflammatory disorders, highlighting risks such as deep vein thrombosis, pulmonary emboli, and herpes virus infections (45, 46). JAK inhibitors should be used cautiously in patients with a history of active malignancy, thromboembolic events, or cardiovascular disease (47). In a pilot study of tofacitinib for refractory BD, two patients with intestinal involvement developed herpes zoster infections, necessitating the discontinuation of the study medication (18). Mild leukopenia and transaminitis, as well as pneumonia, were reported in patients with Behçet’s uveitis treated with upadacitinib (27, 31). To date, no serious adverse events, such as thromboembolism, have been reported in patients with BD treated with JAK inhibitors.

### 3.4 Future directions

Ongoing clinical trials aim to evaluate further the efficacy and safety of JAK inhibitors in BD. Notably, the DRIMID study is investigating filgotinib, a selective JAK1 inhibitor, in patients with refractory BD, idiopathic inflammatory myopathies, and IgG4-related disease (48). This 26-week, open-label phase 2 trial will assess impacts on disease activity and quality of life. Results are eagerly awaited, as they may confirm JAK inhibitors as a viable therapeutic option for BD, a condition historically challenging to manage due to its heterogeneity.

## 4 Conclusion

This case reinforces JAK inhibition as a promising strategy for TNF-α inhibitor-resistant BD, even in the context of dual trisomy 8/9 and MDS. The dose-dependent response and mechanistic correlates underscore the importance of individualized therapy. While preliminary, these findings warrant further investigation through randomized controlled trials to establish standardized protocols for JAK inhibitor use in complex BD phenotypes.

## Data Availability

The original contributions presented in the study are included in the article/supplementary material. Further inquiries can be directed to the corresponding author.
